# Efficiency of an electronic device in controlling tracheal cuff pressure in critically ill patients: a randomized controlled crossover study

**DOI:** 10.1186/s13613-016-0200-2

**Published:** 2016-10-04

**Authors:** Anahita Rouzé, Julien De Jonckheere, Farid Zerimech, Julien Labreuche, Erika Parmentier-Decrucq, Benoit Voisin, Emmanuelle Jaillette, Patrice Maboudou, Malika Balduyck, Saad Nseir

**Affiliations:** 1 Centre de Réanimation, CHU Lille, 59000 Lille, France; 2 Centre d’Investigation Clinique, CHU Lille, 59000 Lille, France; 3 Centre de Biologie et de Pathologie, CHU Lille, 59000 Lille, France; 4 EA 2694 - Santé publique : épidémiologie et qualité des soins, CHU Lille, 59000 Lille, France; 5 Faculté de Pharmacie, Université Lille, 59000 Lille, France; 6 Faculté de Médecine, Université Lille, 59000 Lille, France

**Keywords:** Tracheal cuff, Intubation, Mechanical ventilation, Complications, Microaspiration

## Abstract

**Background:**

Despite intermittent control of tracheal cuff pressure (*P*_cuff_) using a manual manometer, cuff underinflation (<20 cmH_2_O) and overinflation (>30 cmH_2_O) frequently occur in intubated critically ill patients, resulting in increased risk of microaspiration and tracheal ischemic lesions. The primary objective of our study was to determine the efficiency of an electronic device in continuously controlling *P*_cuff_. The secondary objective was to determine the impact of this device on the occurrence of microaspiration of gastric or oropharyngeal secretions.

**Methods:**

Eighteen patients requiring mechanical ventilation were included in this prospective randomized controlled crossover study. They randomly received either continuous control of *P*_cuff_ with Mallinckrodt^®^ device for 24 h, followed by discontinuous control with a manual manometer for 24 h, or the reverse sequence. During the 48 h after randomization, *P*_cuff_ was continuously recorded, and pepsin and alpha amylase were quantitatively measured in tracheal aspirates. *P*_cuff_ target was 25 cmH_2_O.

**Results:**

Clinical characteristics were similar during the two study periods, as well as mean airway pressure. Percentage of time spent with cuff overinflation or underinflation was significantly lower during continuous control compared with routine care period [median (IQR) 0.8 (0.1, 2) vs 20.9 (3.1, 40.1), *p* = 0.0009]. No significant difference was found in pepsin [median (IQR) 230 (151, 300) vs 259 (134, 368), *p* = 0.95] or in alpha amylase level [median (IQR) 1475 (528, 10,333) vs 2400 (1342, 15,391), *p* = 0.19] between continuous control and routine care periods, respectively.

**Conclusions:**

The electronic device is efficient in controlling *P*_cuff_, compared with routine care using a manometer. Further studies are needed to evaluate the impact of this device on intubation-related complications.

*Trial registration* ClinicalTrials.gov Identifier: NCT01965821

## Background

In spite of the increased use of noninvasive ventilation and high-flow nasal oxygen [1–3], intubation is still frequently performed in up to 85 % of critically ill patients requiring mechanical ventilation [4]. This invasive procedure is associated with several potential complications, such as microaspiration of contaminated oropharyngeal and gastric secretions, ventilator-associated pneumonia, and tracheal ischemic lesions [5–10]. These intubation-related complications occur when tracheal cuff is inadequately inflated [11].

Current recommendations are to keep cuff pressure (*P*_cuff_) between 20 and 30 cmH_2_O, using a manometer [12]. Unfortunately, these recommendations are not followed in a high percentage of ICUs [13]. Even when tracheal cuff is routinely monitored and adjusted by nurses, patients spend a large amount of time up to 30–50 % outside the targeted range [14–18]. Moreover, *P*_cuff_ drops under 20 cmH_2_O each time the manometer is connected [19]. Several new devices are available to continuously control *P*_cuff_ and prevent complications related to underinflation or overinflation of tracheal cuff [20–22]. Although many devices are available on the market, few of them were evaluated and validated by well-conducted clinical studies. These devices could be classified into mechanical and electronic. The advantages in using an electronic device are its easy use and the lower cost, compared with a pneumatic device.

The efficiency of the electronic device was evaluated in one in vitro study [23]. However, to our knowledge no clinical randomized controlled study has evaluated the efficiency of the electronic device in critically ill patients receiving mechanical ventilation for more than 48 h. Therefore, we conducted this randomized controlled trial to determine the efficiency of the electronic device in continuously controlling *P*_cuff_. The secondary objective of this study was to evaluate the impact of continuous control of *P*_cuff_, using the electronic device, on microaspiration of gastric contents in intubated critically ill patients.

## Methods

This prospective randomized controlled crossover study was performed during a 1-year period, in a 10-bed ICU at the university hospital of Lille (France), in accordance with the Helsinki Declaration.

### Inclusion and exclusion criteria

Inclusion criteria were: age >18 years and mechanical ventilation through a tracheal tube for a predicted duration of at least 48 h. Exclusion criteria were: mechanical ventilation through a tracheostomy, enrollment in another study that might interfere with the current study results, pregnancy, and contraindication for enteral nutrition.

### Randomization

**Fig. 1:**
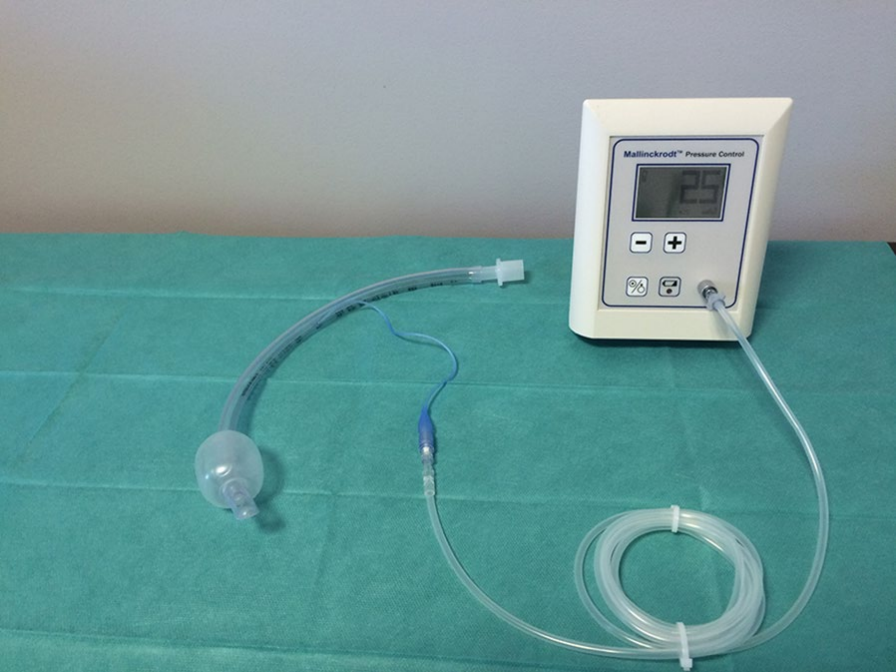
Electronic device connected to a tracheal tube

**Fig. 2:**
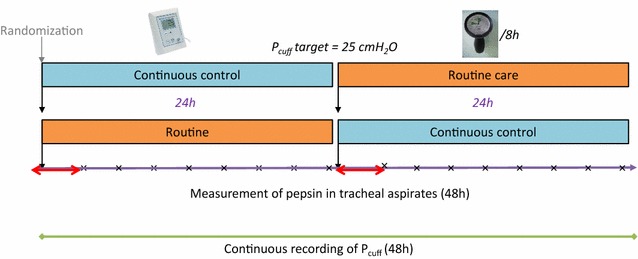
Study design. *Red arrows* indicate washout periods for pepsin and amylase measurement, and *times symbol* indicates each tracheal aspirate performed for pepsin and amylase measurement

Patients were randomly assigned to receive continuous control of *P*_cuff_ with the electronic device (Mallinckrodt electronic cuff pressure controller^®^, VBM Medizintechnik GmbH, Sulz am Neckar, Germany) for 24 h, followed by discontinuous control (every 8 h) with a manual manometer (Hi-Lo Hand Pressure Gauge^®^, Mallinckrodt, Medtronic TM) for 24 h (Fig. 1), or the reverse sequence (Fig. 2). The target of *P*_cuff_ was 25 cmH_2_O during the two periods. Randomization was performed using a computer‐generated random assignment list in balanced blocs of six. Treatment assignments were contained in sealed opaque envelopes sequentially numbered.

### Study objectives and outcome measurement

The primary objective was to determine the efficiency of the electronic device in reducing percentage of time spent with underinflation or overinflation of tracheal cuff, compared with routine care using a manometer. The secondary objectives included the impact of the electronic device on percentage of patients with underinflation or overinflation of tracheal cuff, percentage of time spent with underinflation of tracheal cuff, percentage of time spent with overinflation of tracheal cuff, percentage of time spent with normal (20–30 cmH_2_O) tracheal cuff, *P*_cuff_, and coefficient of variation of *P*_cuff_, compared with routine care, and its impact on microaspiration of gastric and oropharyngeal secretions.

**Fig. 3:**
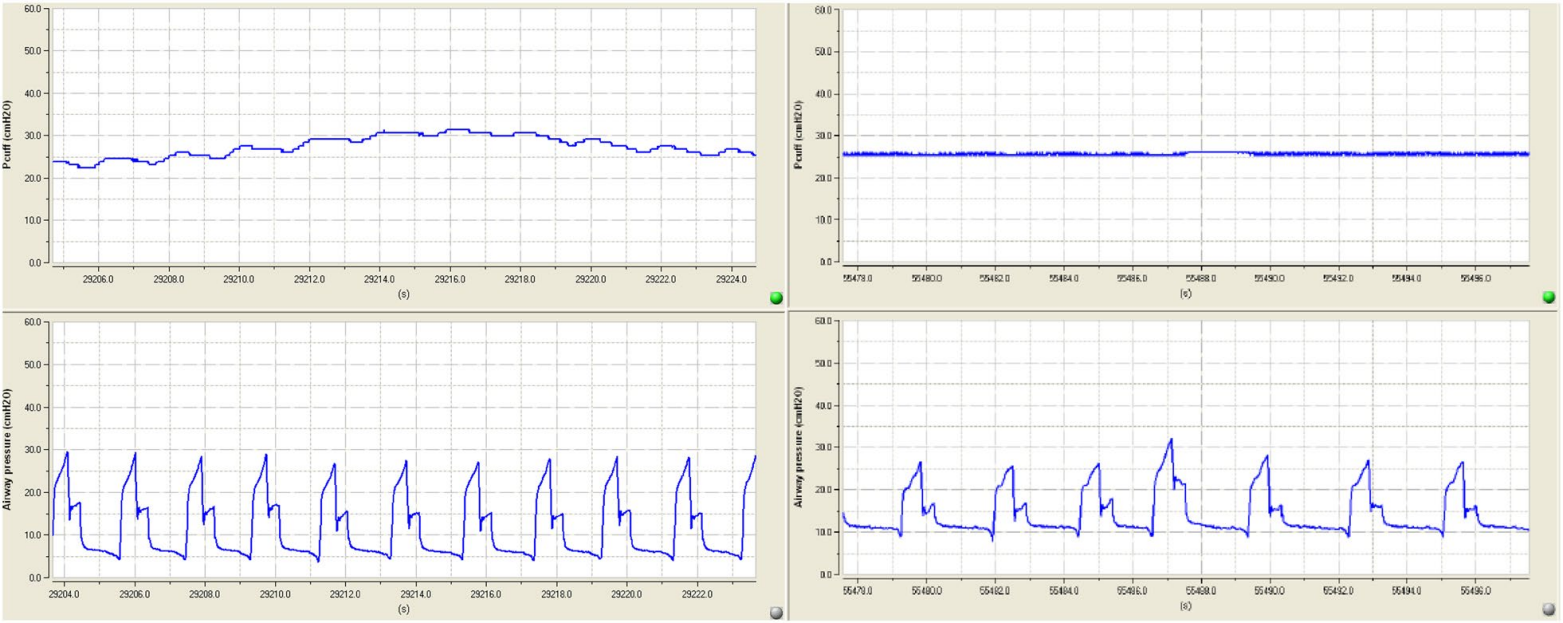
An example of cuff and airway pressure recording. At *left*, cuff pressure (*above*), and airway pressure (*below*), during routine care. At *right*, cuff pressure (*above*) and airway pressure (*below*) during continuous control of cuff pressure

*P*_cuff_ and airway pressure were continuously recorded at a digitizing frequency of 100 Hz for 48 h (Physiotrace^®^; Estaris, Lille, France) [24], including 24 h of continuous control of *P*_cuff_ using the mechanical device and 24 h of manual control of *P*_cuff_ using the manometer (Fig. 3). Pepsin and alpha amylase were quantitatively measured in all tracheal aspirates during the two study periods [25, 26]. In order to avoid interference between the two periods regarding pepsin and alpha amylase levels, tracheal aspirate performed during the first 2 h of each study period was not analyzed. The engineer who analyzed the data (JDJ) and the physicians who measured pepsin and alpha amylase (FZ, PM, and MB) were blinded to study group assignment.

### Study population

All patients were intubated with a high-volume low-pressure PVC standard-cuffed tracheal tube. Tracheal tube size was 8 and 7.5 in men and women, respectively. During the manometer period, nurses adjusted *P*_cuff_ every 8 h. Tracheal suctioning was performed, using open suction system, 6 times a day, or more frequently if clinically indicated. Semi-recumbent position was used during mechanical ventilation. During routine care period, *P*_cuff_ was adjusted, using the manometer, before turning and oral care.

### Definitions

 The primary outcome was the percentage of time spent with underinflation or with overinflation of tracheal cuff. Secondary outcomes included mean *P*_cuff_, coefficient of variation of *P*_cuff_, percentage of patients with underinflation of tracheal cuff, percentage of patients with overinflation of tracheal cuff, percentage of time spent with normal (20–30 cmH_2_O) cuff pressure, percentage of time spent with underinflation of tracheal cuff, percentage of time spent with overinflation of tracheal cuff, mean pepsin and alpha amylase level, percentage of tracheal aspirates positive for pepsin, and percentage of tracheal aspirates positive for alpha amylase.

Underinflation of tracheal cuff was defined as *P*_cuff_ <20 cmH_2_O for >5 min over the 24-h period of recording. Overinflation of tracheal cuff was defined as *P*_cuff_ >30 cmH_2_O for >5 min over the 24-h period of recording [14]. The coefficient of variation of *P*_cuff_ was calculated as standard deviation/mean *P*_cuff_ × 100.

Microaspiration of gastric contents was defined by the presence of pepsin at significant level (>200 ng/mL) in tracheal aspirate. Microaspiration of oropharyngeal secretions was defined by the presence of alpha amylase at significant level (>1685 UI/L) in tracheal aspirate [26].

### Statistical analyses

#### Sample size calculation

Based on previous results [14, 15], the mean percentage of time spent with underinflation or overinflation of tracheal cuff was 30 % [standard deviation (SD) = 20 %] in patients intubated with a PVC‐cuffed tracheal tube receiving routine care of *P*_cuff_ using a manual manometer. The expected mean percentage of time with underinflation or overinflation of tracheal cuff using the mechanical device was 10 % (expected difference of 20 %). In a parallel-group design, *n* = 22 patients per group will be required to detect this difference with a two-sided test, a power of 90 %, an alpha risk of 5 %, and a SD of 20 %. In a crossover design, the sample size determination is based on SD within subject difference, which can be derived from SD of measure and coefficient correlation (*r*) between the two measures on the same subject [27]. The sample size can therefore be derived from the number of patients to be included in parallel-group design, as follows: *n* * (1 − *r*). Thus, assuming a conservative value of 0.2 for *r*, the number of patients to include is 18.

#### Result analysis

All analyses were performed in an intention-to-treat manner. Distribution of quantitative variables was tested using Shapiro–Wilk test. Normally and non-normally distributed variables were expressed as mean ± SD and median (25th, 75th interquartile), respectively. The statistical significance was set at *p* < 0.05.

The primary outcome was compared using a mixed linear model, adjusting for the period effect. Interaction between study period and assigned treatment, i.e., continuous control of *P*_cuff_ or routine care, was tested. Qualitative and quantitative patient characteristics and secondary outcomes were compared between the two 24-h periods using McNemar and Wilcoxon tests, respectively.

## Results

**Fig. 4:**
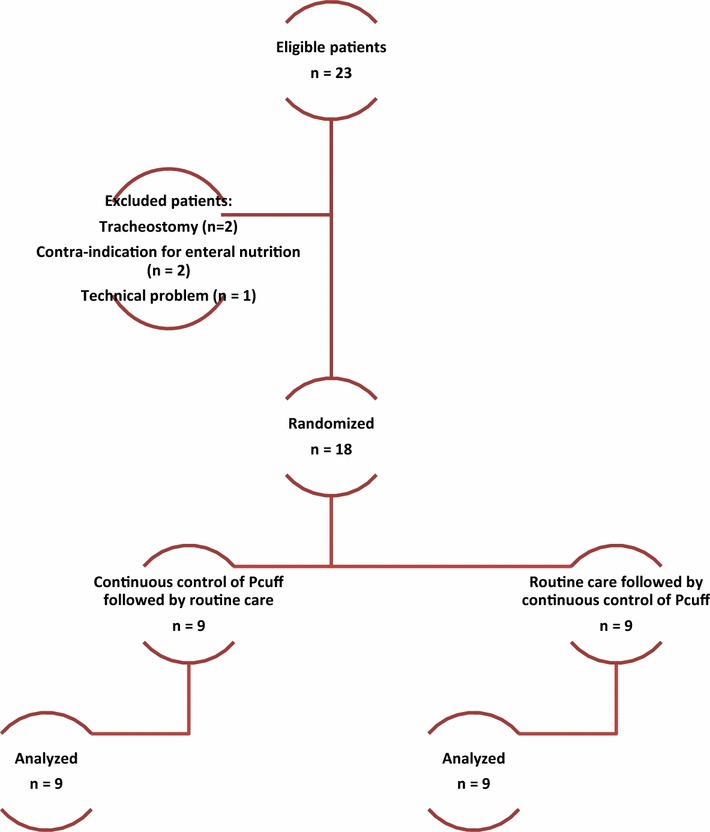
Flowchart

During the study period, 23 patients were eligible. Five patients were excluded for different reasons, and 18 patients were included and were all analyzed (Fig. 4).

### Patient characteristics

Patient characteristics are presented in Table 1.

**Table 1:** Patient characteristics

Number of included patients	18
Age (years), mean ± SD	54 ± 18
Male gender, *n* (%)	13 (72)
Weight (kg), median (IQR)	72 (66, 81)
SAPS II, mean ± SD	43 ± 16
LOD score, mean ± SD	6 ± 3
Cause for ICU admission, *n* 5 %	
Neurologic failure	7 (38)
Respiratory failure	6 (33)
Shock	5 (27)
Duration of mechanical ventilation before inclusion (days), median (IQR)	4 (2, 6)
Total duration of mechanical ventilation (days), median (IQR)	12 (7, 25)
Length of ICU stay (days), median (IQR)	21 (11, 30)
Ventilator-associated pneumonia, *n* (%)	3 (15)
ICU mortality, *n* (%)	10 (55)

*SAPS* Simplified Acute Physiology Score, *LOD* logistic organ dysfunction, *IQR* interquartile range

No significant difference was found between the two study periods regarding ventilator mode and settings, sedation, Ramsay score, or neuromuscular blocking agent use. Prone position was not used in included patients, during the two study periods. All other characteristics were also similar during the two periods (Table 2).

**Table 2:** Patient characteristics during the 48 h following randomization

Variables	Continuous control of *P*_cuff_ *n* = 18	Routine care *n* = 18	p
Ventilatory mode^a^			>0.99
ACV	13 (72)	13 (72)	
PSV	9 (50)	10 (56)	
Mean *P*_peak_ (cmH_2_O)	31 (21, 35)	30 (24, 34)	0.64
Mean PEEP (cmH_2_O)	7 (5, 9)	6 (5, 8)	0.73
Mean FiO_2_	0.45 (0.40, 0.50)	0.40 (0.40, 0.50)	0.41
Number of tracheal suctions	6 (5, 7)	6 (6, 7)	>0.99
Sedation	12 (67)	14 (78)	0.50
Ramsay score	5 (3, 6)	4 (3, 6)	0.86
Neuromuscular blocking agent use	1 (6)	0 (0)	>0.99
Head-of-bed position <30°	4 (22)	6 (33)	0.50
Fiberoptic bronchoscopy	2 (11)	2 (11)	>0.99
Transport outside the ICU	0 (0)	1 (6)	>0.99
Aerosolized medication	2 (11)	2 (11)	>0.99
_*i*_NO	2 (11)	1 (6)	>0.99
Quantity of enteral nutrition (mL/day)	1000 (500, 1500)	1000 (875, 1125)	0.31
Gastric residual volume (mL)	0 (0, 61)	0 (0, 100)	0.52
Vomiting	2 (11)	2 (11)	>0.99
Prokinetic drugs	1 (6)	1 (6)	>0.99
Stress ulcer prophylaxis or treatment	16 (89)	16 (89)	>0.99

Data are number of patients (%) or median (Interquartile range)

*P*_*cuff*_ cuff pressure, *ACV* assist–control ventilation, *PSV* pressure support ventilation, *P*_*peak*_ peak pressure, *PEEP* positive end-expiratory pressure, *FiO*_*2*_ fraction of inspired oxygen, _*i*_ *NO* inhaled nitric oxide

^a^Some patients received two ventilator modes during continuous control of *P*_cuff_, or during routine care

No significant difference was found in duration of *P*_cuff_ and airway pressure recording [median (IQR) 23 (23, 23.3 vs 23.5 h (23, 24), *p* = 0.066], or in mean airway pressure [13.2 (10.7, 15.5) vs 13.1 cmH_2_O (10.8, 15.6)] between continuous control and routine care periods, respectively.

### Primary outcome

The percentage of time spent with underinflation, or with overinflation, was significantly lower during continuous control of *P*_cuff_ compared with routine care [median (IQR) 0.8 (0.1, 2) vs 20.9 (3.1, 40.1), *p* = 0.0009]. No significant interaction was found between study period and the assigned treatment (*p* = 0.91).

### Secondary outcomes

Mean *P*_cuff_ and percentage of time spent with *P*_cuff_ 20–30 cmH_2_O were significantly higher during continuous control of *P*_cuff_ compared with routine care. Percentage of patients with underinflation, percentage of time spent with underinflation, percentage of time spent with overinflation, and coefficient of variation of *P*_cuff_ were significantly lower during continuous control compared with routine care of tracheal cuff. Percentage of patients with overinflation was similar during the two study periods (Table 3).

**Table 3:** Secondary outcomes

Variables	Continuous control *n* = 18	Routine care *n* = 18	*p*
Mean *P*_cuff_ (cmH_2_O)	25.9 (25.5, 26.4)	22.7 (21.6, 24.8)	0.001
Coefficient of *P*_cuff_ variation (%)	4.1 (1.5, 6.1)	7.7 (4.4, 11.5)	<0.001
*P*_cuff_ 20–30 cmH_2_O			
Yes	18 (100)	18 (100)	>0.99
% of recording time	99.1 (97.9, 99.9)	73.5 (54.4–96.8)	<0.001
*P*_cuff_ <20 cmH_2_O			
Yes	0 (0)	13 (72)	<0.001
% of recording time	0.0 (0, 0)	9.1 (0.0–36.1)	0.001
*P*_cuff_ >30 cmH_2_O			
Yes	11 (61)	14 (78)	0.25
% of recording time	0.8 (0.1, 2.1)	2.4 (0.7, 9.4)	<0.001
Pepsin (ng/mL)	230 (151, 300)	259 (134, 369)	0.95
% of tracheal aspirates positive for pepsin	67 (0, 100)	71 (0, 100)	0.83
Alpha amylase (ng/mL)	1475 (528, 10,333)	2400 (1342–15,391)	0.19
% of tracheal aspirates positive for alpha amylase	40 (0, 100)	33 (0, 100)	0.92

Data are number of patients (%) or median (interquartile range)

*P*_*cuff*_ cuff pressure

Yes indicates that a patient had the variable at least once

No significant difference was found in pepsin level, in percentage of tracheal aspirates positive for pepsin, in alpha amylase level, or in percentage of tracheal aspirates positive for alpha amylase between the two study periods (Table 3).

## Discussion

Our results suggest that the electronic device is efficient in controlling *P*_cuff_. However, no significant impact of continuous control of *P*_cuff_ was found on microaspiration of gastric or oropharyngeal secretions.

To our knowledge, our study is the first clinical randomized controlled study to evaluate the efficiency of the electronic device in continuously controlling *P*_cuff_ in critically ill patients. A previous in vitro study found similar results [23]. In addition, Lorente et al. [28] conducted a prospective observational study to determine the impact of continuous control of *P*_cuff_, using the same electronic device, on the incidence of VAP. The authors reported significantly lower rate of *P*_cuff_ determinations <20 cmH_2_O (mean ± SD 0 vs 9 ± 8, *p* < 0.001), *P*_cuff_ determinations >30 cmH_2_O (mean ± SD 0 vs 4 ± 5, *p* < 0.001), and substantial decrease (51 %) in VAP incidence in patients who received continuous control of *P*_cuff_, compared with those received routine care. However, the efficiency of the electronic device in continuously controlling *P*_cuff_ was not the primary objective of the study. In addition, *P*_cuff_ was not continuously recorded, and the study was not randomized.

The percentage of time spent with underinflation (median 9.1 %) and with overinflation (median 2.4 %) during routine care was lower than previously reported [14, 15]. The small number of patients included in the current study (*n* = 18) could explain this difference. Duration of prior intubation was identified as an independent risk factor for underinflation of tracheal cuff [14]. However, duration of prior intubation was quite similar in the current study (median 4 days) compared with previous studies (2 and 5 days, respectively) [14, 15]. Another explanation could be the different patient characteristics between the current study and previous ones. The percentage of non-sedated patients was lower in the current study (22 %) compared with the two previous ones (35 and 53 %, respectively). The absence of sedation was identified as an independent risk factor for underinflation of tracheal cuff [14].

No significant impact of continuous control of *P*_cuff_ was found on microaspiration of gastric and oropharyngeal secretions. Several explanations could be suggested for this finding. Microaspiration of gastric contents was a secondary outcome, and our study was probably underpowered to detect such an effect. The higher, but not significant, level of alpha amylase during continuous control, compared with routine care periods, is in keeping with this hypothesis. Although this secondary outcome was negative, the results could be helpful for future studies, aiming at evaluating the impact of continuous control of *P*_cuff_ on microaspiration. In addition, the duration of tracheal aspirate collection (24 h) during the two study periods was probably too short to evaluate this effect. Further, the above-mentioned in vitro study reported that the electronic device might interfere with self-expanding properties of some PVC-cuffed tracheal tubes [23]. Therefore, the rapid correction of overinflation of *P*_cuff_ during cough could result in short sudden drop of *P*_cuff_ and microaspiration of gastric contents. Further studies are needed to confirm this hypothesis. The short washout period (2 h) used in this study might have resulted in overlap in pepsin and alpha amylase results between the two periods. Whilst pepsin half-life is relatively short [29], alpha amylase half-life is unknown. This might have influenced the impact of continuous control of *P*_cuff_ on microaspiration of gastric and oropharyngeal secretions.

Given the efficiency of the electronic device, the absence of potential harm, and the reduction in nurse workload, one could argue that use of such a device could be recommended in every intubated critically ill patient. However, the level of evidence on the clinical benefit of using continuous control of *P*_cuff_ is still low [30]. In addition, cost-effectiveness of this preventive measure of VAP was not evaluated. Therefore, further randomized controlled trials aiming at evaluating the impact of continuous control of *P*_cuff_ on VAP incidence are required to evaluate the efficiency of this preventive measure.

Some limitations of our study should be acknowledged. First, this was a single-center study. Therefore, our results could not be generalized to patients hospitalized in other ICUs. We did not evaluate the impact of continuous control of *P*_cuff_ using the electronic device on ventilator-associated pneumonia or tracheal ischemic lesions. However, our study design did not allow such an evaluation, because each patient was his own control. This design is probably the best first step to evaluate the efficiency of the electronic device, because of potential patient-related confounders, such as tracheal size, shape, respiratory resistance, and airway pressure. Further, the study was not blinded. However, investigators who assessed continuous *P*_cuff_ recording and pepsin were blinded to study group assignment.

## Conclusions

The electronic device evaluated in this study is efficient in continuously controlling *P*_cuff_ in critically ill patients. Further randomized controlled studies are needed to determine the impact of continuous control of *P*_cuff_, using the electronic device, on intubation-related complications, such as microaspiration, VAP, and tracheal ischemia.

## Abbreviations

*P*_cuff_: cuff pressure
SAPS: Simplified Acute Physiology Score
LOD: logistic organ dysfunction
